# Supplementary figures and images for: Divergent genetic mechanism leads to spiny hair in rodents
Source: PLoS One. 2018 Aug 17;13(8):e0202219. doi: 10.1371/journal.pone.0202219 (PMC6097693; doi:10.1371/journal.pone.0202219)

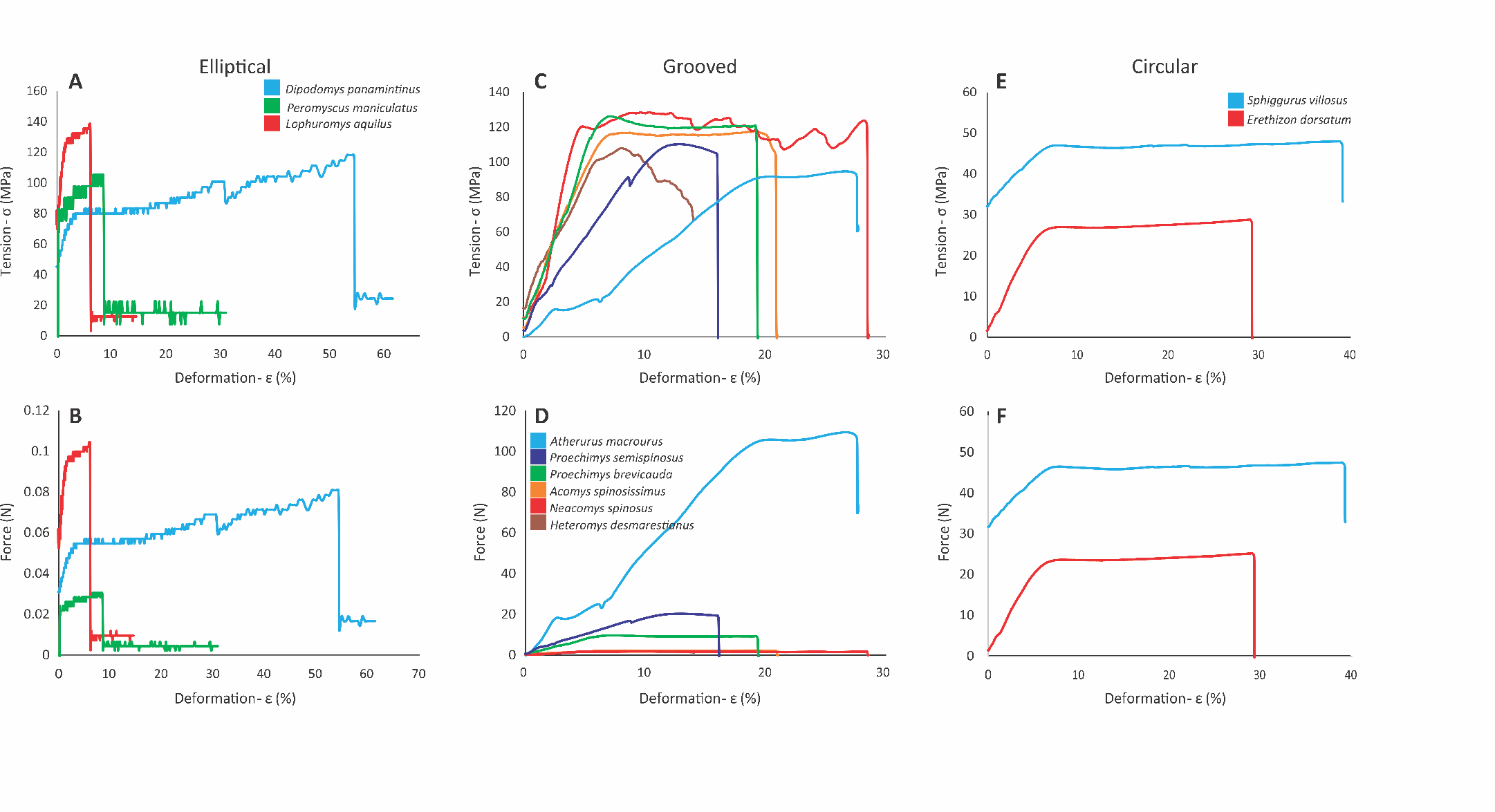

Supplement: S1 Fig — Deformation curves in relation to tension (A, C, E) and force (B, D, F) for hairs of eleven rodent species, clustered according to the cross-section shape of the hair. (TIF) [file pone.0202219.s001.tif]

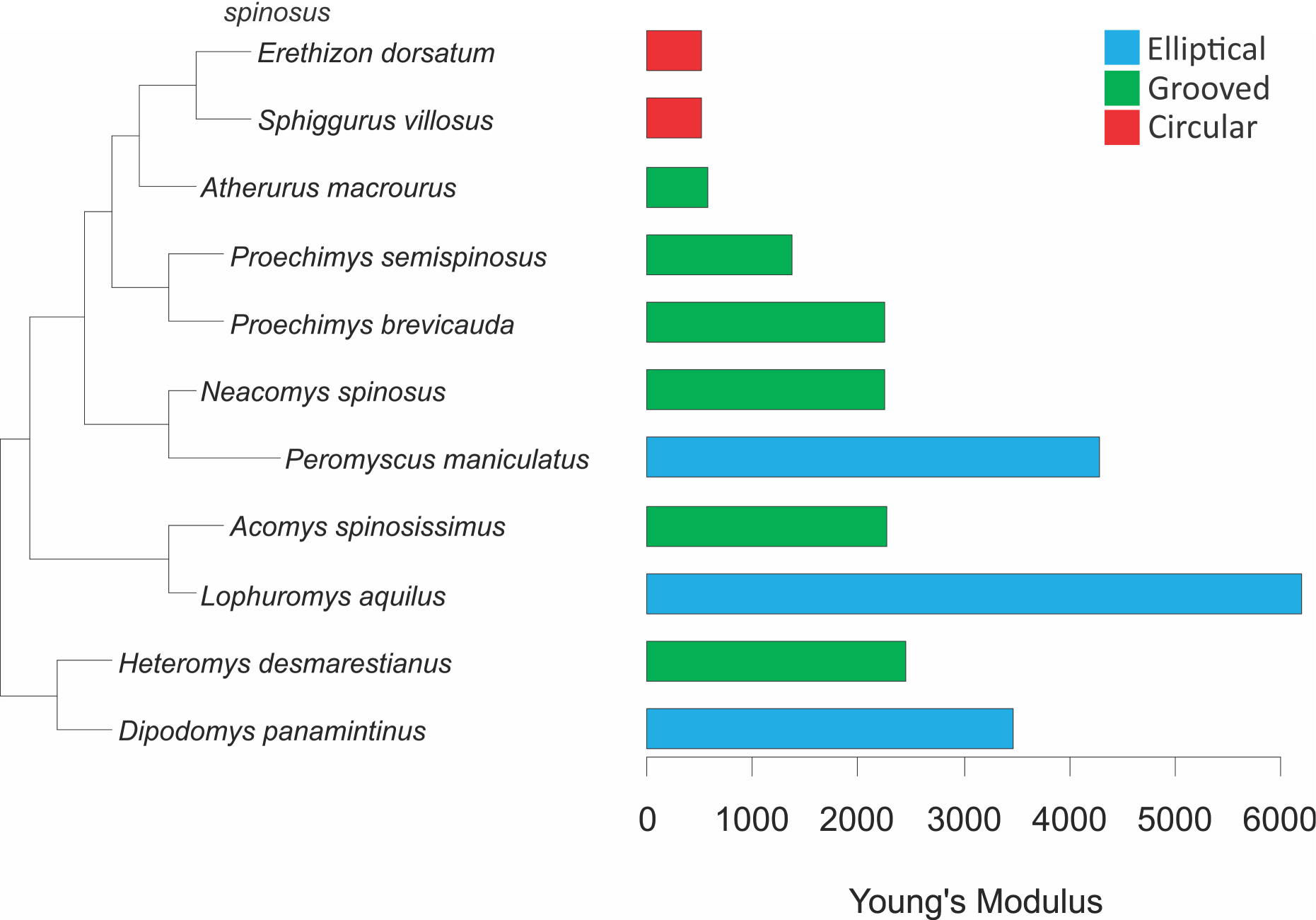

Supplement: S2 Fig — The modulus was calculated as E = σ/ε for the initial portion of each deformation curve (at 3% of deformation). (TIF) [file pone.0202219.s002.tif]
